# Mandatory adherence to diagnostic protocol increases the yield of CTPA for pulmonary embolism

**DOI:** 10.1007/s13244-016-0509-2

**Published:** 2016-07-22

**Authors:** Stefan Walen, Erwin de Boer, Mireille A. Edens, Corné A. J. van der Worp, Martijn F. Boomsma, Jan Willem K. van den Berg

**Affiliations:** 1 Department of Pulmonology, Isala, Dr. van Heesweg 2, 8025 AB Zwolle, The Netherlands; 2 Department of Radiology, Isala, Dr. van Heesweg 2, 8025 AB Zwolle, The Netherlands; 3 Clinical Epidemiologist, Isala, Dr. van Heesweg 2, 8025 AB Zwolle, The Netherlands

**Keywords:** Pulmonary embolism, CTPA (computed tomography pulmonary angiography), Diagnostic yield, Diagnostic protocol, Protocol adherence

## Abstract

**Objectives:**

To determine if mandatory adherence to a diagnostic protocol increases the rate of computed tomography pulmonary angiographies (CTPAs) positive for pulmonary embolism (PE)—the so-called diagnostic yield. Further, we aim to identify factors associated with this diagnostic yield.

**Methods:**

We included all patients with suspected PE requiring CTPA from 9 January 2014 t0 3 June 2014. The requesting physicians were forced to follow diagnostic workup for PE by calculating a Wells score and, if necessary, determining D-dimer level. The percentage of positive CTPA scans was calculated and compared with our previous cohort (Walen et al. Insights Imaging 2014;5(2):231–236). Odds ratios were calculated as a measure of association between dichotomous variables and CTPA findings.

**Results:**

Of 250 scans, 74 were positive (29.6 % [95 % CI, 24.3-35.5 %]) and 175 were negative (70 %). The percentage positive scans increased with 6.6 % and the percentage negative scans decreased with 3.1 %. This change was statistically significant (*p* = 0.001). Independent clinical predictors of diagnostic yield were previous deep venous thrombosis (DVT) (OR, 3.22; *p* = 0.013) and clinical signs of DVT (OR, 2.71; *p* = 0.012). Chronic obstructive pulmonary disease (COPD) was negatively associated with PE (OR, 0.33; *p* = 0.045).

**Conclusions:**

This study shows that mandatory adherence to a diagnostic protocol increases the yield of CTPA for PE in our centre.

***Main Messages*:**

• *Mandatory adherence to diagnostic protocol increases the yield of CTPA for PE*

• *Previous DVT and signs of DVT were associated with a higher yield*

• *No patients with a low Wells score and a low D-dimer had PE*

## Introduction

Computed tomography pulmonary angiography (CTPA) is widely used for confirming the diagnosis of pulmonary embolism (PE). With a sensitivity between 60 and 100 % and a specificity between 81 and 98 %, it has replaced pulmonary angiography as the diagnostic reference standard [1–4]. Although effective, CT scanning exposes patients to ionising radiation. Furthermore, administration of intravenous radiocontrast can lead to acute kidney injury, especially in high-risk patients. Therefore, first an assessment of clinical probability should be made to determine the likelihood of being able to confirm the diagnosis of PE by imaging. The most commonly used method to predict pre-test probability is using the Wells algorithm [5]. First, a Wells score should be calculated to determine the clinical probability. Secondly, either the Wells score is high (a score of more than four points) and a CTPA should be performed or, if the Wells score is low (four points or less), the D-dimer level should be assessed. Thirdly, a high D-dimer level (≥0.5 μg/mL) also dictates performing CTPA. This diagnostic management strategy was prospectively validated in a large Dutch cohort and has proved effective and safe [6]. However, implementation in daily practice seems to be difficult. A previous study conducted in our teaching hospital in The Netherlands showed poor documented adherence to diagnostic protocol [7]. As an example, Wells scores, which should be calculated for every patient suspected of PE, were only reported in 13 % of cases. We hypothesised that better protocol adherence would lead to a higher rate of CTPA positive for PE—the so-called diagnostic yield. Therefore, we conducted our current study, in which we aimed to improve diagnostic yield of CTPA by forcing doctors to write down their clinical observations, Wells score and D-dimer on the request form. Our further objective was to find clinical factors associated with diagnostic yield.

## Materials and methods

In this prospective observational study, all patients with suspected PE requiring a CTPA scan in the period from 9 January 2014 to 3 June 2014 were included. Approval of the local ethics committee was received. All data were acquired using a Philips 256-slice Brilliance iCT or 128-slice Ingenuity CT scanner (Philips Healthcare, Best, The Netherlands). Peak voltage was 100 kV with an exposure of 250 mAs per slice. Slice thickness was 0.625 mm for both scanners. Tube rotation time was 0.5 for the 128-slice scanner and 0.4 for the 256-slice scanner with a pitch of 0.798 and 0.696, respectively. The scans were reconstructed with a soft tissue filter (filter B) containing a window width (WW) of 150 and a window level (WL) of 90. To display the pulmonary arteries, 60-75 mL intravenous contrast fluid (Optiray™ 350) was administered with a flow of 4.0–5.0 mL/s. The pulmonary arteries were scanned in the early arterial phase, triggered on the arrival of contrast fluid in the main pulmonary artery with a threshold of HU >150 to start the scan. We used iDose (variable levels) for iterative image reconstruction. Every CTPA scan was read double-blinded by two experienced radiologists with an expertise in chest imaging. When there was a difference in interpretation consensus was sought. When the quality of the scan was so low that it could not be stated whether there was a pulmonary embolism or not, a scan was called undiagnostic. As an intervention every physician in our hospital requesting a CTPA for pulmonary embolism was asked to document Wells-scores on the request form and—if available—to document D-dimer. Special templates of the request form with a pre-printed Wells scoring table were distributed among requesting physicians. When the required information was lacking on the request forms our diagnostic radiographers urged the requesting doctor to provide the necessary clinical data. However, no scans were refused. If a scan was nevertheless performed without the clinical data documented on the request form, the scores were retrospectively obtained. Electronic and paper medical files were searched for clinical characteristics and relevant medical history. We compared the patient data in our current study with patient data in our previous, 2011 cohort [7].

Data are summarised in tables and graphs. Categorical data are presented as *n* (%), and tested using the Fisher’s exact test. Confidence intervals were calculated for dichotomous variables [25]. Continuous data were tested for normality using the Shapiro-Wilk test, in addition with plots. Continuous and ordinal data are presented as median (1st–3rd quartile), and were tested using the Mann-Whitney *U* test. Univariate and multivariable logistic regression analysis was used for finding associates of CTPA-diagnosed pulmonary embolism. In addition, diagnostic indices were calculated and receiver operating characteristic (ROC) analysis was performed. Our present study was compared to our previous study by means of multivariable logistic regression analysis with ‘CTPA outcome’ as dependent variable, and ‘study’ with potential confounders as independent variables. All tests and confidence intervals were performed two-tailed, using alpha 5 % as significance level.

## Results

### Study population and CTPA scans

A total of 250 patients underwent CTPA scanning in our hospital. Of 250 scans, 74 were positive (29.6 % [95 % CI, 24.3-35.5 %]), 1 was undiagnostic (0.4 %) and 175 were negative (70 %). Tables 1 and 2 show patient characteristics and a subgroup comparison of patients with a positive versus a negative scan. In summary, age, history of deep venous thrombosis (DVT), signs of DVT, Wells score and D-dimer were significantly different between patients with a positive scan versus patients with a negative scan. Chronic obstructive pulmonary disease (COPD), Wells category and groundglass appearance were borderline significantly (*p* < 0.1) different between patients with a positive scan versus patients with a negative scan. Table 3 shows univariate and multivariable logistic regression analysis for associates of positive CTPA scan. In summary, history of DVT, signs of DVT, Wells score and Wells category were positively associated with CTPA-diagnosed pulmonary embolism; whereas COPD was negatively associated with CTPA-diagnosed pulmonary embolism. Both age and groundglass appearance were borderline (*p* < 0.1) positively associated with CTPA-diagnosed pulmonary embolism.

**Table 1:** Patient characteristics and subgroup comparison of patients with a positive CTPA versus a negative CTPA scan

Patient characteristics	All patients (*n* = 250)	Subgroup comparison		
		CTPA positive (*n* = 74)	CTPA negative (*n* = 175)	*p* value
Origins				
ER Hospital Outpatient clinic	155 (62 %) 87 (34.8 %) 8 (3.2 %)	49 (66.2 %) 24 (32.4 %) 1 (1.4 %)	105 (60 %) 63 (36 %) 7 (4 %)	0.543
Demographics				
Sex (women)	134 (53.4 %)	40 (54.1 %)	93 (53.1 %)	1.000
Age (years)	64 (49–73)	66 (52.5–77)	62 (46–71)	0.044*
Clinical risk factors				
Immobilisation (yes)	40 (16 %)	12 (16.2 %)	28 (16 %)	1.000
Paresis/paralysis legs or cast in the past 4 weeks (yes)	10 (4 %)	3 (4.1 %)	7 (4 %)	1.000
Surgery past 4 weeks (yes)	25 (10 %)	11 (14.9 %)	14 (8 %)	0.110
Trauma (yes)	6 (2.4 %)	0 (0 %)	6 (3.4 %)	0.183
History of DVT (yes)	21 (8.4 %)	11 (14.9 %)	9 (5.1 %)	0.019*
History of PE (yes)	14 (5.6 %)	5 (6.8 %)	9 (5.1 %)	0.564
COPD (yes)	30 (12 %)	4 (5.4 %)	26 (14.9 %)	0.053**
Heart failure under treatment (yes)	20 (8 %)	5 (6.8 %)	15 (8.6 %)	0.800
Pacemaker (yes)	3 (1.2 %)	0 (0 %)	3 (1.7 %)	0.557
Active malignancy (yes)	64 (25.6 %)	17 (23 %)	47 (26.9 %)	0.634
Central venous catheter (yes)	2 (0.8 %)	1 (1.4 %)	1 (0.6 %)	0.507
Oestrogen use women (yes)	*n* = 134 women 3 (2.2 %)	*n* = 40 women 1 (2.5 %)	*n* = 93 women 2 (2.2 %)	1.000
Pregnant women (yes)	*n* = 134 women 7 (5.2 %)	*n* = 40 women 0 (0 %)	*n* = 93 women 7 (7.5 %)	0.102
Clinical factors				
Dyspnea (yes)	182 (72.8 %)	54 (73 %)	128 (73.1 %)	1.000
Chest pain (yes)	116 (46.4 %)	31 (41.9 %)	84 (48 %)	0.406
Signs of DVT (yes)	30 (12 %)	15 (20.3 %)	15 (8.6 %)	0.017*
Haemoptysis (yes)	11 (4.4 %)	3 (4.1 %)	8 (4.6 %)	1.000
Syncope (yes)	12 (4.8 %)	3 (4.1 %)	9 (5.2 %)	1.000
Hypotension (syst < 100) (yes)	8 (3.2 %)	3 (4.1 %)	5 (2.9 %)	0.698
Tachycardia (>100/bpm) (yes)	80 (32 %)	29 (39.2 %)	51 (29.1 %)	0.138
Tachypnea (>30/min) (yes)	10 (4 %)	4 (5.4 %)	6 (3.4 %)	0.489
Hypoxaemia (Sp/aO2 < 90 %) (yes)	44 (17.6 %)	14 (18.9 %)	30 (17.1 %)	0.720
Hypothermia (<36gr centigrade) (yes)	2 (0.8 %)	0 (0 %)	2 (1.1 %)	1.000
Altered consciousness (yes)	2 (0.8 %)	0 (0 %)	2 (1.1 %)	1.000
Wells score	4 (3–4.5)	4.5 (3–6)	4 (3–4.5)	0.008*
Wells category (>4)	124 (49.6 %)	44 (59.5 %)	79 (45.1 %)	0.052**
D-dimer - <0.5 - ≥0.5	*n* = 159 12 (7.5 %) 147 (92.5 %)	*n* = 50 0 (0 %) 50 (100 %)	*n* = 109 12 (11 %) 97 (89 %)	0.019*
Pulmonary infiltrate (yes)	*n* = 249 37 (14.9 %)	7 (9.5 %)	30 (17.1 %)	0.171
Atelectasis (yes)	*n* = 249 52 (20.9 %)	13 (17.6 %)	39 (22.3 %)	0.496
Consolidation (yes)	*n* = 249 34 (13.7 %)	13 (17.6 %)	21 (12 %)	0.312
Groundglass (yes)	*n* = 249 18 (7.2 %)	9 (12.2 %)	9 (5.1 %)	0.062**

*p < 0.05, significant

**p < 0.1, borderline significance

For one female patient the scan was undiagnostic. Total number of positive and negative CTPA and imaging findings add up to *n* = 249

*CTPA* computed tomography scanning of the pulmonary arteries, *ER* emergency room, *DVT* deep venous thrombosis, *PE* pulmonary embolism, *COPD* chronic obstructive pulmonary disease, *Syst* sytolic

*Immobilisation* recently bedridden for more than 3 days or paralysis, paresis or plaster immobilisation of the leg

*Trauma* any major injury, regardless of region of impact and all minor injuries involving the extremities

*Active malignancy* malignancy with treatment within the last 6 months or palliative care or best supportive care

**Table 2:** Pulmonary embolism by Wells category and D-dimer category

Pulmonary embolism	All patients (*n* = 249 CTPA scans)					
- None	175 (70.3 %)					
- Proximal	64 (25.7 %)					
- Subsegmental	10 (4 %)					
Pulmonary embolism	Wells low			Wells high		
	*n* = 126 (50.6 %)			*n* = 123 (49.4 %)		
- None	96 (76.2 %)			79 (64.2 %)		
- Proximal	27 (21.4 %)			37 (30.1 %)		
- Subsegmental	3 (2.4 %)			7 (5.7 %)		
	D-dimer low	D-dimer unknown	D-dimer high	D-dimer low	D-dimer unknown	D-dimer high
	*n* = 7 (5.6 %)	*n* = 29 (23 %)	*n* = 90 (71.4 %)	*n* = 5 (4.1 %)	*n* = 61 (49.6 %)	*n* = 57 (46.3 %)
Pulmonary embolism						
- None	7 (100 %)	26 (89.7 %)	63 (70 %)	5 (100 %)	40 (65.6 %)	24 (59.6 %)
- Proximal	-	3 (10.3 %)	24 (26.7 %)	-	17 (27.9 %)	20 (35.1 %)
- Subsegmental	-	-	3 (3.3 %)	-	4 (6.6 %)	3 (5.3 %)

High Wells score: >4 points

High D-dimer level: ≥0.5 μg/mL

*CTPA* computed tomography scanning of the pulmonary arteries

**Table 3:** Logistic regression analysis for associates of CTPA-diagnosed pulmonary embolism

	Univariate logistic regression		
	β	*p* value	OR (95 % CI)
Origins			
Total	−	0.463	−
Hospital Outpatient clinic	−0.203 −1.184	0.493 0.274	0.816 (0.457–1.458) 0.306 (0.031–2.557)
Demographics			
Sex (women)	0.037	0.895	1.037 (0.601–1.789)
Age (years)	0.017	0.054**	1.017 (1.000–1.034)
Clinical risk factors			
Immobilisation (yes)	0.016	0.966	1.016 (0.486–2.127)
Paresis/paralysis legs or cast in the past 4 weeks (yes)	0.014	0.984	1.014 (0.255–4.034)
Surgery past 4 weeks (yes)	0.697	0.105	2.008 (0.865–4.659)
History of DVT (yes)	1.170	0.013*	3.220 (1.274–8.142)
History of PE (yes)	0.290	0.614	1.337 (0.432–4.132)
COPD (yes)	–1.116	0.045*	0.327 (0.110–0.974)
Heart failure under treatment (yes)	–0.258	0.631	0.773 (0.270–2.210)
Active malignancy (yes)	–0.208	0.522	0.812 (0.430–1.535)
Central venous catheter (yes)	0.869	0.541	2.384 (0.147–38.623)
Oestrogen use women (yes)	0.154	0.901	1.167 (0.103–13.247)
Clinical factors			
Dyspnea (yes)	–0.009	0.978	0.991 (0.537–1.829)
Chest pain (yes)	–0.247	0.377	0.781 (0.451–1.352)
Signs of DVT (yes)	0.998	0.012*	2.712 (1.249–5.889)
Haemoptysis (yes)	–1.126	0.856	0.882 (0.227–3.422)
Syncope (yes)	–0.249	0.715	0.779 (0.205–2.964)
Hypotension (syst <100) (yes)	0.362	0.626	1.437 (0.334–6.173)
Tachycardia (>100/bpm) (yes)	0.449	0.122	1.567 (0.887–2.769)
Tachypnea (>30/min) (yes)	0.476	0.471	1.610 (0.441–5.879)
Hypoxaemia (Sp/aO2 <90 %) (yes)	0.120	0.737	1.128 (0.559–2.276)
Wells score	0.219	0.005*	1.245 (1.067–1.453)
Wells category (>4)	0.578	0.040*	1.782 (1.027–3.093)
CT findings			
Pulmonary infiltrate (yes)	–0.683	0.125	0.505 (0.211–1.208)
Atelectasis (yes)	–0.297	0.404	0.743 (0.370–1.491)
Consolidation (yes)	0.447	0.245	1.563 (0.736–3.317)
Groundglass (yes)	0.938	0.057**	2.554 (0.971–6.720)

*p < 0.05, significant

**p < 0.1, borderline significance

*CTPA* computed tomography scanning of the pulmonary arteries, *DVT* deep venous thrombosis, *PE* pulmonary embolism, *COPD* chronic obstructive pulmonary disease

### Diagnostic accuracy

**Fig. 1:**
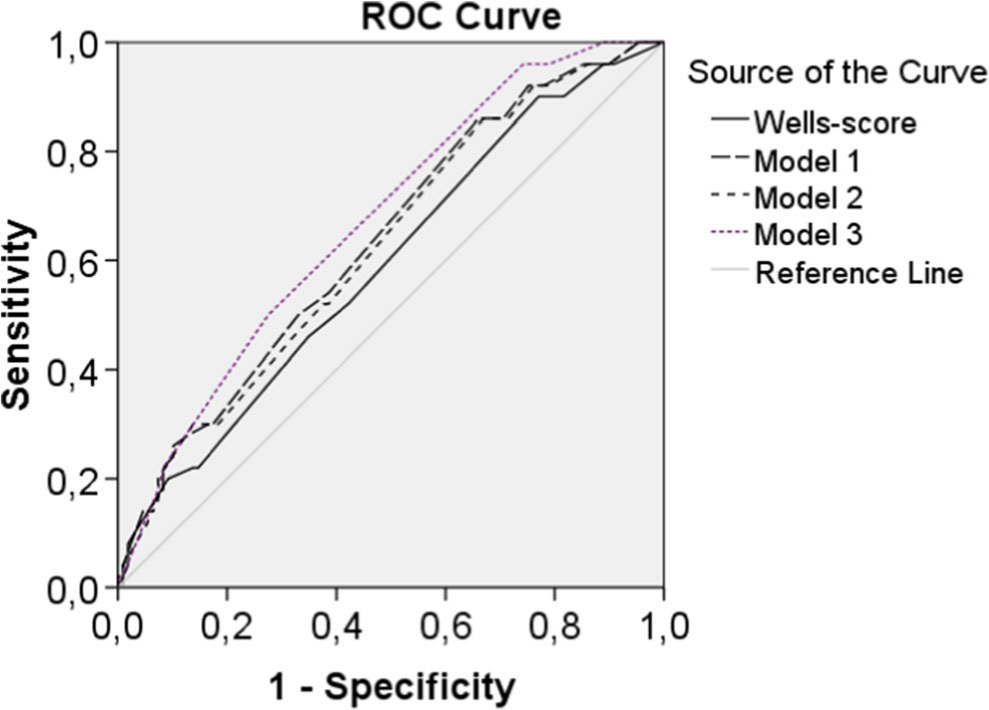
Diagnostic accuracy of Wells score and models

Table 4 shows sensitivity and specificity of Wells category, D-dimer category, history of DVT and signs of DVT. In an attempt to improve diagnostic accuracy, several multivariable models were examined. Diagnostic accuracy of Wells score compared with the multivariable models is visualised using ROC analysis, as shown in Fig. 1.

**Table 4:** Diagnostic accuracy

	CTPA-diagnosed PE		
	Yes	No	
Wells category			
Wells >4	44	79	**123 (PPV, 38.8 %)**
Wells ≤4	30	96	**126 (NPV, 76.2 %)**
Total	74 (SE, 59.5 %)	175 (SP, 54.9 %)	
D-dimer category (*n* = 159)			
D-dimer high	50	97	**147 (PPV, 34 %)**
D-dimer low	0	12	**12 (NPV, 100 %)**
Total	50 (SE, 100 %)	109 (SP, 11 %)	
History of DVT			
History of DVT (yes)	11	9	**20 (PPV, 55 %)**
History of DVT (no)	63	166	**172 (NPV, 72.5 %)**
Total	74 (SE 14.9 %)	175 (SP, 94.9 %)	
***Signs of DVT***			
Signs of DVT(yes)	15	15	**30 (PPV, 50 %)**
Signs of DVT (no)	59	160	**219 (NPV, 73.1 %)**
	74 (SE, 20.3 %)	175 (SP, 91.4 %)	

*NPV* negative predictive value, *PE* pulmonary embolism, *PPV* positive predictive value, *SE* sensitivity, *SP* specificity, *DVT* deep venous thrombosis

### Comparison with previous results

Table 5 shows the change in CTPA scan findings (raw data) compared to our previous study (Walen et al. [7]). The percentage of positive scans has increased with 6.6 %, the percentage of undiagnostic scans has decreased with 3.5 %, and the percentage of negative scans has decreased with 3.1 %. This change was statistically significant (*p* = 0.001). When ignoring undiagnostic scans, there was a borderline (*p* < 0.1) significant increase in positive scans (*p* = 0.070). We compared both study populations in order to find potential confounders. We looked at a difference in origin of patients (ER, hospital or outpatient) and compared the clinical variables that were associated with CTPA outcome in either the current multivariable analysis and/or the analysis of our first cohort, notably sex, age (positive), COPD (negative), cardiac history (negative) and dyspnoea (positive). A comparison of origins of patients revealed a significant difference (ER, 72 % vs 62 %; hospital, 22.5 % vs 34 %; outpatient clinic, 4.9 % vs 3.2 %) for the previous and the present study respectively (*p* < 0.001). A comparison of age revealed a significant difference (median 66 years vs 64 years) for the previous and the present study respectively (*p* = 0.022). A comparison of cardiac history revealed a significant difference of 26 % vs 8 % for the previous study and the present study respectively (*p* < 0.001). Logistic regression analysis with CTPA-outcome as dependent variable and study as independent variable, using the previous study as reference, revealed a borderline significant *p* value (0.062). Adjustment of study for origin improved this *p* value to 0.056. Adjustment of study for age improved this *p* value to *p* = 0.043. Adjustment of study for both origin and age improved this *p* value to 0.036. However, adjustment of study for cardiac history deteriorated this *p* value to *p* = 0.153. Adjustment of study for origin, age and cardiac history, resulted in a non-significant adjusted *p* value (0.113), with an adjusted odds ratio of 1.298 and 95% CI of 0.940–1.791]).

**Table 5:** Change in CTPA scan findings

	Walen et al. 2014 [7]			Present study			Difference (%)
	*n*	%	95 % CI	*n*	%	95 % CI	
Positive	224	23 %	20.5 -25.8 %	74	29.6 %	24.3-35.5 %	**6.6 %**
Undiagnostic	38	4 %	2.3-5.3 %	1	0.4 %	0.1–2.2 %	**−3.5 %**
Negative	712	73.1 %	70.2-75.8 %	175	70 %	64.1–75.3 %	**−3.1 %**
Total	974			250			

*CTPA* computed tomography scanning of the pulmonary arteries

## Discussion

There is ample evidence supporting the use of pre-test probability rules to help decide if a CTPA should be ordered in a patient in whom PE is suspected. Of these, the Wells score is arguably the most commonly used [5, 6]. However, numerous studies have shown that protocol adherence in clinical practice is poor [7–9]. The objective of this study was to investigate if we could increase diagnostic yield of CTPA for pulmonary embolism by influencing the behaviour of requesting physicians. As an intervention, we forced doctors to write down Wells scores and, if available, the D-dimer on the CTPA request form for all patients they suspected of PE. We used our 2011 cohort with a total diagnostic yield of 23.0 % as a reference standard. Of the 249 patients that were scanned 29.6 % had PE. This is relatively high in comparison with values reported in the literature (6.7–31 %) [10–15]. Another study in The Netherlands found a diagnostic yield of 19.1 % [16]. What’s more important, when directly compared with our 2011 cohort, the sole intervention of making doctors write down the relevant clinical data led to an increase in positive CT scans by 6.6 %. The overall percentage of isolated subsegmental pulmonary emboli was 4 % and was not different from our 2011 cohort. Seven patients (3 %) had a low Wells score and a low D-dimer level. None of these patients had a PE, thereby confirming the safety of the refraining from performing a CTPA in this group. Clinical characteristics of our cohort resemble existing data; dyspnoea and chest pain were the most common presenting symptoms, active malignancy and immobilisation were the most frequently found risk factors [17, 18]. In patients both with and without PE CTPA showed a high percentage of consolidation (respectively 18 % and 12 %) and atelectasis (18 % and 22 %). A previous study by Akram et al. [19] showed similar rates. A history of DVT and current signs of DVT were both significantly associated with a higher diagnostic yield of PE. This finding is consistently found in the literature [20–23] and accentuates the importance of including these risk factors when establishing the clinical probability of PE. Both variables are included in the Wells-score. The presence of COPD was associated with a lower diagnostic yield. The fact that COPD and PE share their cardinal symptom, dyspnoea, could be the explanation for this association. Unsurprisingly, both a high Wells score and a high D-dimer value were also associated with a higher diagnostic yield.

To our knowledge, this is the first study that shows that the diagnostic yield of CT scanning can be improved by influencing requesting behaviour. In 2007 Albrizio and Mizzi [24] published their study of a comparison of positive rates of CTPA before and after implementation of the Wells score in their diagnostic protocol. No significant difference was found. However, it was not clear if the new protocol was in fact adequately applied in clinical practice, making interpretation of the results difficult. Another study, conducted by Kanaan et al. [8], evaluated if an educational intervention had an effect on appropriateness rates and outcomes of CTPA. No difference was found, showing that it takes more to improve protocol adherence than a single educational intervention. A more recent study tried to improve utilisation and CTPA outcome by mandatory assignment of the Wells score to an electronic request form [9]. What was remarkable was that requesting physicians appeared to inflate Wells scores over time (in spite of the fact that no threshold score was required to perform a CTPA), leading to an increase in appropriate use of CTPA, but failing to improve the positive rate for PE. We did not observe this effect, which can explain why an increase in appropriate use of CTPA did increase the percentage of positive scans in our study. If our results can be reproduced in other studies it has direct implications for daily practice. It is a strong plea to first establish the clinical probability of a disease before progressing to imaging techniques.

### Limitations

Our study has its limitations. With a total of 249 patients our current cohort is quite small. Further, we did not randomise between an intervention group and a control group, but compared our current cohort with historical control data, which can lead to bias. However, when we compared our two cohorts we used the raw data, and we statistically adjusted for pre-diagnostic differences between both study populations. There were no changes in clinical assessment of the patients between our current cohort and our previous one. Furthermore, because all CT scans were blinded and double read by the same qualified radiologists as in our previous cohort, difference in reader experience could not be the reason for the increase in the rate of positive scans. What did differ was the type of scanners used. In our current cohort we used 128- and 256-slice CT scanners, set to a maximum potential energy of 100 kV. In our 2011 cohort, 64-slice scanners with a 120 kV protocol were used. It is possible that because of an increase in diagnostic accuracy very small emboli were better visualised, but the percentage of isolated subsegmental emboli in both studies was low and equal (4 %). There was in fact a decrease in the number of non-diagnostic studies from 4 to 0.4 %. However, because all undiagnostic scans were of very poor quality in both cohorts (severe breathing artefacts or almost no radiocontrast in the pulmonary artery) it is less likely that the type of scanner used contributed much to this difference. Lastly, in the few cases a scan was made without a documented Wells score on the request form, the score was calculated in retrospect, which could lead to bias because the result of the scan (positive or negative for PE) was known by the investigator calculating the score.

## Conclusions

Our study shows that the sole intervention of asking doctors to provide adequate and relevant clinical data seems to improve the diagnostic yield of CTPA for PE. These results should be further analysed in large prospective trials. Our data also confirm that it is absolutely safe to refrain from scanning patients with a low Wells score and a low D-dimer level as none of the scanned patients in this subgroup had CTPA-diagnosed PE.
